# A novel online training programme for healthcare professionals caring for older adults

**DOI:** 10.1007/s40520-023-02464-1

**Published:** 2023-06-22

**Authors:** Jean-Pierre Michel, Fiona Ecarnot, Hidenori Arai, Liang-Kung Chen

**Affiliations:** 1 University of Geneva, Geneva, Switzerland; 2 EA3920, University of Franche-Comté, Besançon, France; 3 National Center for Geriatrics and Gerontology, Obu, Japan; 4 Center for Health Longevity and Aging Sciences, National Yang Ming Chiao Tung University College of Medicine, Taipei, Taiwan

**Keywords:** Education, Geriatrics, Gerontology, Healthcare workers, Ageing

## Abstract

The proportion of older people in the world population is growing rapidly. Training and retaining healthcare professionals in sufficient numbers in the field of ageing represents a major challenge for the future, to deal with the healthcare needs of this ageing population. The COVID pandemic has unfortunately compounded shortages of healthcare workers worldwide. There is therefore a pressing need to scale-up the education of healthcare professionals in geriatrics and gerontology. Over the last 30 years, a group of motivated geriatrics physicians from Europe have been striving to educate healthcare professionals in geriatrics and gerontology through various initiatives, and using innovative pedagogic approaches to train physicians, nurses and other healthcare professionals around the world. The COVID-19 pandemic unfortunately put a stop to presence-based training programmes, but prompted the development of the online International Association of Gerontology and Geriatrics (IAGG) eTRIGGER (e-Training In Geriatrics and GERontology) course, a new training course in geriatrics and gerontology for healthcare professionals from a wide range of backgrounds. We outline here the history of the educational initiatives that have culminated in the roll-out of this new programme, and the perspectives for the future.

## Background

Caring for the ever-increasing population of older adults will be one of the most challenging medical and public health issues of the twenty-first century. Attracting, training, and retaining sufficient numbers of healthcare professionals in the field of ageing will have a tremendous impact on the world population. It will respond to an urgent and inescapable need, will help decrease political tensions, and should mitigate the global care burden and increase the well-being of the older population, whose number is increasing rapidly and at unprecedented speed.

These issues have been brewing on the horizon for decades, but are now become quite alarming for two major reasons. The first is the accelerating growth of the world population of adults aged over 60. The second is due to a foreseeable shortage of skilled healthcare workers, which was not adequately anticipated and was markedly compounded by the COVID pandemic.

Regarding the growth of the older population, the world recently passed a demographic landmark, as we now have more adults aged over 65 years than children under the age of 5 [1]. The current population of 900 million adults aged ≥ 65 worldwide looks set to increase to around 2.1 billion by 2050, doubling its share of the total population to 16% [2]. Moreover, projections for the developing world, which will account for 80% of the 2.1 billion adults over 65 in 2050, are particularly alarming [3].

One of the main reasons population ageing is such an issue is the speed at which the proportion of adults over 65 will move from 10 to 20% of the overall population in each country. In France, it took 120 years for the proportion of over 65 s to increase from 10 to 20% of the whole population, whereas it will take only 40 years in Japan, and only 30 years in Brazil, China, and India [4]. These countries are not prepared to cope with the new needs of the increasing older population, and this will have direct repercussions for those most in need of care, and at risk of losing functional independence and autonomy. In most countries in the world, the increase in longevity is unfortunately associated with a longer amount of time spent in dependency, i.e., in a state requiring help for the activities of daily living from family carers or healthcare professionals, regardless of the place of residence (home or institutional care). Furthermore, linked to the sharp decrease in natality, there is a marked transition from large families to nuclear families in all world countries [5]. This phenomenon is compounded by migration from rural to urban areas and/or from low-income to high-income countries [6, 7]. The parents and grandparents who are left behind will rapidly need more professional assistance and care [8].

These demographic and societal phenomena, as well as world health data, justify the urgent need for more healthcare professionals, to ensure appropriate care for the rapidly ageing population. However, there is a global shortage of healthcare professionals [9], which underscores the compelling need to improve knowledge and skills in geriatrics and gerontology among healthcare professionals, and to ensure there are adequate numbers of professionals qualified to deliver care.

Comparing the current stock of healthcare workers to the minimum levels required to meet a target score of 80 on the Universal Health Coverage (UHC) effective service coverage index, researchers estimated that there is a shortage of more than 43 million health and care workers, including 30.6 million nurses and midwives and 6.4 million physicians [3]. Numerous factors have been shown to contribute to healthcare worker shortages, including the out-migration of health workers, war and political unrest, violence against healthcare workers, and insufficient incentives for training and retention. The COVID pandemic has unfortunately accelerated this unfavourable trend quite sharply. In the USA, since the pandemic began in early 2020, one in five healthcare workers has quit their jobs, and surveys suggest that up to 47% of healthcare workers plan to leave their positions by 2025 [10]. The World Health Organization has referred to this situation as a “ticking timebomb” and proposes a list of 10 actions to strengthen the health and care workforce [11]. Of note, half of the proposals concern ways to improve the education of healthcare workers. The proposals include aligning education with population needs and health service requirements, strengthening professional development, expanding the use of digital tools to support the workforce, building leadership capacity, and increasing public investment in workforce education. Other suggested measures include developing strategies to recruit and retain healthcare workers in rural and remote areas, creating working conditions that promote a healthy work-life balance and protecting the mental health of the workforce, improving health information systems for better data collection and analysis, and optimizing public funding investments to promote innovative workforce policies.

Clearly, there is a pressing need to intensify the education of future academic leaders (doctors, nurses, and other healthcare workers) in the field of geriatrics and gerontology. Enhanced education will update their knowledge, improve their skills, and enable them to better use communication and education technologies for the benefit of their vulnerable, aged patients. Moreover, this skilled workforce will enhance the empowerment of ageing adults, by helping to control their midlife risk factors, and thereby contribute to delaying many preventable age-related diseases [12]. This in turn can avert or delay entry into dependency, and enable larger numbers of elders to live the latter period of their life with dignity, and free of pain [13]. Therefore, educating healthcare professionals adequately, to ensure they are present in sufficient numbers to care for the increasing population of elders, will help achieve better outcomes in these elders, with wide-ranging benefits all across society in a virtuous circle.

## Training in geriatrics: a 30-year history

In the early 1990s, geriatric medicine was still in its infancy, and was often neglected, being considered a “secondary” specialty, although some countries were pioneering in the field. Indeed, the Italian Society of Gerontology and Geriatrics (SIGG), one of world's oldest, founded in Florence in 1950, was officially acknowledged by the International Association of Gerontology and Geriatrics (IAGG) as early as 1950. Against this background, four motivated key opinion leaders in geriatric medicine in Europe, namely Professor Hannes Staehelin (Switzerland), Sir John Grimley Evans (UK), Professor Jean-Pierre Michel (Switzerland), and Professor Bertil Steen (Sweden) decided together to found the “European Academy for Medicine of Ageing” (EAMA), with the aim of training future leaders in geriatric medicine. Undoubtedly, this philanthropic initiative was ground-breaking at the time, bearing in mind that in the 1990s, no high-level teaching activities existed in the field of medicine of ageing.

These colleagues set up the EAMA with the overarching aim of training specialists in geriatric medicine to a high standard of knowledge, and to equip them to train other physicians and healthcare collaborators in their turn, thereby exponentially increasing the potential for education in geriatric medicine [14–16]. The tremendous success of this initiative attracted many promising participants from Europe, and from farther afield. Once they went back to their home countries and their careers flourished successfully, several alumni decided to create new branches of the EAMA around the world, notably the Latin America Academy for Medicine of Ageing (ALMA) in 2002, the Middle East Academy for Medicine of Ageing (MEAMA) in 2004, and the Asia Academy of Medicine of Ageing (AAMA) in 2011. In each region, the training programme based on the original EAMA model was culturally adapted to local conditions, and was often opened not only to physicians, but also to other healthcare professionals caring for older adults in their countries [17].

The EAMA programmes use an innovative new method of teaching and training, namely the “reverse” teaching method or “flipped learning”, by assigning homework to the students before the course [18]. The idea is to involve the participants in the teaching programme by asking them to prepare, present and defend lectures, discuss culturally-based clinical cases in small group discussions, imagine themselves managing a geriatric ward, coping with a public health crisis, or arguing with decision-makers about the importance of caring for older populations, without neglecting applications for external collaborative research grants. This form of teaching model gives the learners a leading role while increasing and updating their knowledge and enhancing their professional skills. Moreover, the worldwide origins of the trainees enable them to establish strong and lasting professional networks. All this is particularly relevant in today’s context, and we can retrospectively affirm that these training programmes were not only beneficial for the participants, but also for the volunteer tutors who rose to the new educational challenges and prospective ideas. Moreover, it has been the origin of numerous international and cross-cultural research papers [19, 20].

These international presence-based training sessions were recognized by the International Association of Gerontology and Geriatrics—World (IAGG-W) executive board, which decided to set up and fund the IAGG Federation of Geriatric Education (IAGG FGE) in 2017. In this way, the activities of the existing Academies of Medicine of Aging were partly financed for the first time since their creation. This led to the creation of two academies of geriatric medicine: the first (and sole) training session of the Southeast Asia Academy of Geriatrics (SEAAG) was held in Bangkok in 2018. Unfortunately, the second project, namely the creation of the African Academy of Geriatrics (AAMEG) in 2020, had to be cancelled due to the COVID pandemic.

**Fig. 1:**
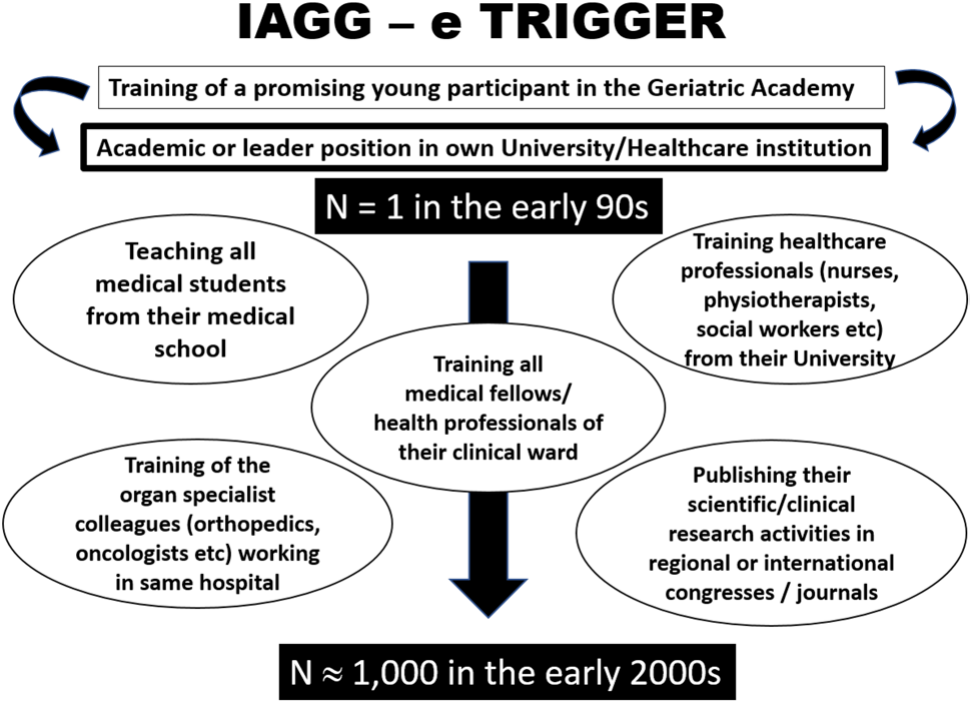
The snowball effect of training the worldwide future leaders in the care of older population. Highly selected participants are taught to spread their new acquired knowledge and skills to numerous other medical students, physicians, and health care professionals, to compensate for the shortfall in skilled healthcare professionals. It is estimated that within 10 years, each awardee from any IAGG Academy of Medicine of Aging would teach/train about 1000 medical students, colleagues and healthcare professionals

The global demand for these courses, and the persistently high rates of attendance and success, have confirmed the compelling need for continuing professional education in geriatrics around the world. The promotion of former participants of the different Academies of Geriatrics to positions as professors of geriatric medicine in their respective countries has been highly appreciated and marks the success of this training initiative. As each successful alumnus teaches other colleagues and healthcare professionals, the benefits filter through caring communities around the world, with a knock-on beneficial effect on the quality of care for aged populations. Starting in the 1990s, the training activities continued until the COVID-19 pandemic brought a halt to all presence-based activities. Nevertheless, the long-standing popular success of these teaching programmes amplified their global beneficial impacts on care (Fig. 1). Former participants who advanced to high-grade positions in their respective countries subsequently acted as tutors during master-classes on ageing. Currently, the leaders of the four existing Academies of Geriatrics are former laureates of the programme themselves, which testifies to the quality of the training and the passion of the teaching they received.

## The COVID-19 pandemic and the need for a change in the paradigm

However, in early 2020, the world was plunged into the throes of the emerging COVID-19 pandemic, which put a sudden stop to all existing presence-based initiatives, and aborted the development of the African project for an Academy of geriatrics. As telecommunication, teleconsultations and video-conferences started to be part of the new daily life, it was time to review how to continue and expand on the previously acquired experience in training in geriatrics. Given that the younger generations were born and grew up with technological development, online teaching immediately stood out as the only way forward. Thus, the IAGG eTRIGGER programme was born, a one-year teaching programme for healthcare professionals of all horizons involved in the care of older adults. The first session of the new programme took place in December 2021, and the first year’s programme had over 70 registered trainees, of whom 37 successfully obtained their diplomas in December 2022 after the first 12 months of teaching.

The first major difficulty in implementing the IAGG eTRIGGER programme was finding a reliable technical and academic platform capable of supplying and managing the kind of online course we would like to develop and at an affordable price. The potential cost of developing two websites de novo (a website for the public with general information about the course, and a dedicated teaching platform for communicating with the learners) was heatedly discussed since skilled manpower would be needed for such an ambitious project. Thankfully, the Health Science e-Training Foundation (HSeT: https://hset.org) met all the prerequisites, and provides excellent online resources for students and teachers alike.

A second dilemma that was a direct corollary of the first problem above, related to the financial sustainability of the training. The IAGG Federation of Geriatric Education set up in 2017 by Prof. John Rowe, then head of the IAGG-W enjoyed the luxury of a budget funded by profits from the San Francisco IAGG-W congress. The delayed organisation of the Buenos Aires IAGG-W congress due to the COVID pandemic, and the conversion to a virtual event, dashed hopes of anticipated financial profits. However, thanks to the IAGG-W’s solid commitment to pursuing and extending training for healthcare professionals in the care of older adults, the IAGG eTRIGGER programme continues, with experts participating on a voluntary basis, while, starting from the second year of the programme (January 2023), a minimal financial participation is requested of the students.

A third strategic choice in the organisation of the IAGG eTRIGGER programme, was to target countries in urgent need of training in the care of older adults. In this regard, a key target quickly became apparent: Asian countries represent the most populous part of the world, and their populations are ageing the fastest. This decision was also simplified due to the excellent collaboration with, and success of the Asian Academy of Geriatrics, shared by Profs. Hidenori Arai (Japan) and Liang-Kung Chen (Taiwan). The earlier presence-based training sessions organised in Asian and Southeast Asian countries clearly demonstrated the emergence of young geriatricians willing to rise to positions of academic authority.

Another key question was to decide to open the training to all health professionals (physicians and non-physicians) who were already experienced in the care of older adults, and with high leadership potential. This proposal seemed self-evident, given the fundamental importance of multidisciplinary teamwork in providing quality care for older adults [21]. This decision implied involving existing leaders in gerontology (nursing, psychology, rehabilitation, and sociology but also experts in dentistry, engineering, pharma etc.) as teachers. This new perspective raised interesting and innovative ideas, with the accompanying challenge of opening the minds of clinicians, who too often overlook the environmental, psycho-social, and economic components of their patients’ lives. In this domain, more so than in the medical domain, close collaboration with worldwide experts appeared useful.

**Fig. 2:**
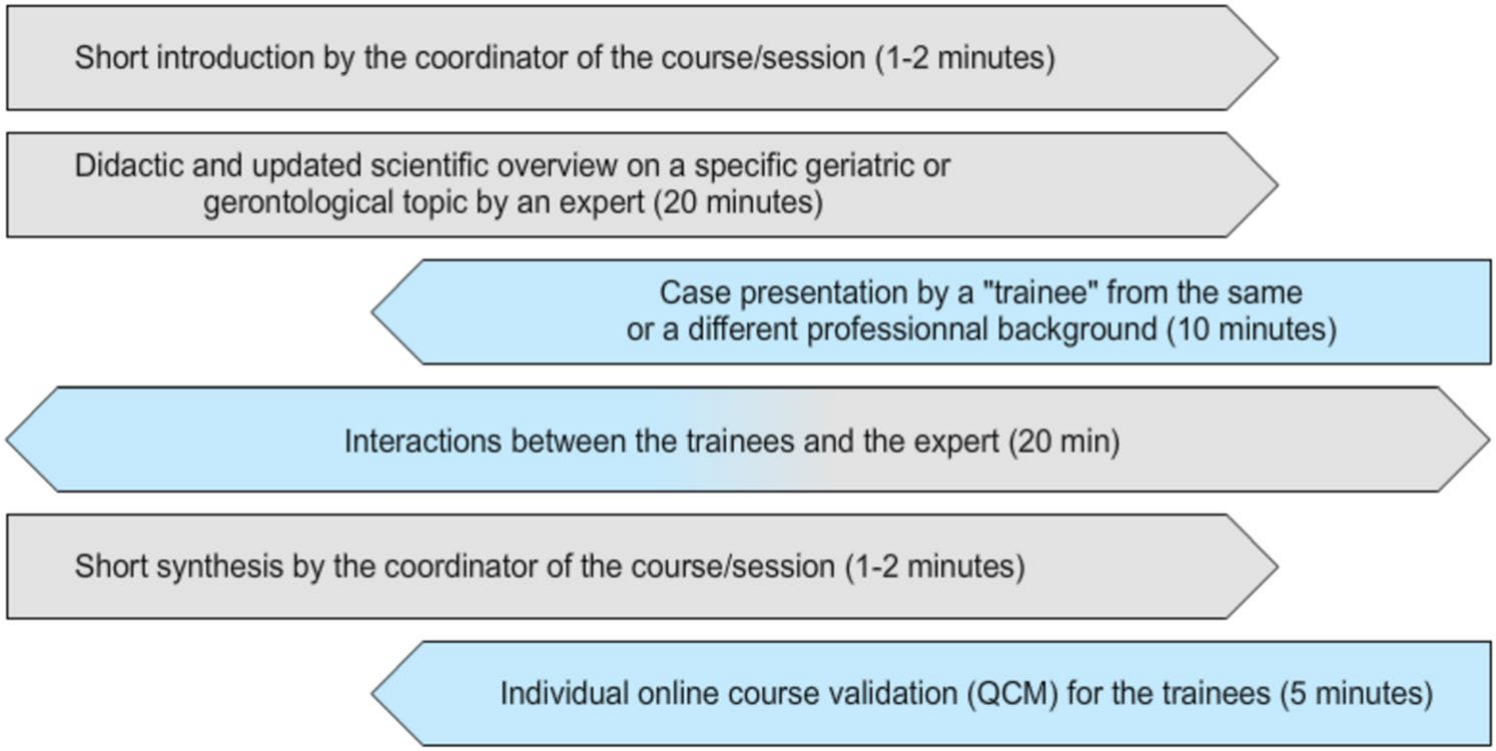
Format of the IAGG e-TRIGGER sessions. This format, covering 1 h, is used during each hour of the monthly 3-h session of multidisciplinary teaching on a predefined topic

Lastly, a new and interactive model of training needed to be imagined. It was consensually decided to apply a model inspired by the ECHO training method [22, 23], which combines key lectures, culturally adapted clinical case presentations, and general group discussion on the topic, before finishing with key take-home messages by the lead tutor (Details can be found on the IAGG-FGE website: https://iagg-fge.org/). Each course comprises 12 monthly sessions of 3 h. The most important issue is keeping the momentum for three hours of online teaching, bringing together complementary medical, nursing, socio-psychological, managerial/economic or therapeutic approaches, without neglecting the cultural background of the cases. This difficult exercise is shared out between renowned experts (called “tutors”) for the keynote lectures, academic healthcare professionals with proven clinical leadership skills (called “scholars”), and the learners registered for the programme (called “trainees”) (Fig. 2). Each three-hour session is comprised of three blocks of one hour, further subdivided into a 20-min lecture by a Tutor, followed by a 10-min case presentation by a scholar, then 30 min of moderated discussion with all the Trainees. Each monthly session addresses an important topic in geriatric medicine or gerontology, such as healthy ageing, frailty, sarcopenia, gerontechnology, end-of-life issues, and others.

There was a concern that the delivery of the course in English would represent a barrier, and restrict the discussion, but it quickly became apparent that the language barrier was not prohibitive, with the Trainees eager to participate and uninhibited by the language. Indeed, the main motivation of the trainees is not only to acquire new knowledge, but also to enhance their skills and understand differences in care and management between countries.

The annual IAGG eTRIGGER programme was established in harmony between the leaders of the regional IAGG e-TRIGGER course. The tutors were chosen by the Asian colleagues, and if local experts in their region were unavailable, international tutors were chosen to give the keynote lectures. The tutors prepare didactic lectures on major geriatric or gerontological topics, with one specific topic being the focus of each monthly session. Tutors also prepare multiple choice questions based on their key messages, to enable the trainees to validate their participation via a continuing medical education quiz, made available on the teaching platform after each session. Scholars are also chosen from the region, to present culturally adapted cases from their clinical practise, reflecting the main issues in their countries. This gives rise to interesting and enriching interprofessional exchanges, and raises awareness of the similarities and differences between countries.

Finally, each monthly session receives accreditation from the European Accreditation Council for Continuing Medical Education (EACCME®) of the Union Européenne des Médecins Spécialistes (UEMS; European Union of Medical Specialists). This accreditation is valid in Europe, and North and South America. Unfortunately, no equivalent continental-level accreditation could be found for the Asian countries. Trainees who successfully pass ten of the twelve CME quizzes held during the year receive an IAGG-W certificate of participation. In December 2022, a total of 37 laureates from 9 Asian countries obtained this valuable award among the trainees who registered for the first year’s course, testifying to the attractiveness of the IAGG e-TRIGGER certification.

## Future developments for the IAGG e-TRIGGER programme

The plenary lecture devoted to the IAGG e-TRIGGER initiative during the 2022 IAGG-W congress immediately raised significant interest in other countries not yet involved in the programme. Requests came in from Australia, India, and Turkey almost immediately. The executive team organising the Asia IAGG e-TRIGGER integrated new delegates to expand the reach and extend the collaboration of the existing team (Prof. Hidenori Arai (Japan), Prof. Liang-Kung Chen (Taiwan)) with colleagues from Australia (Prof. Julie Byles), Mainland China (Prof. Sofia Lin Kang), and India (Prof. Ashish Goel and Prof. Arvind Mathur). The 2023 IAGG e-TRIGGER course, which started its second year in January 2023, was renamed “ASIO” for Asia and Oceania IAGG e-TRIGGER. The number of participants has increased substantially over the first year, despite the introduction of fees, and the involvement of the previous year’s successful trainees as scholars has intensified the discussions of the culturally targeted clinical cases. Indeed, the same system of teaching, accreditation, and continuing medical education examination is being reiterated.

More promising developments for the IAGG e-Trigger are afoot. Prof. Mario Barbagallo and Dr. Radhouane Gouiaa, respectively Presidents of the IAGG European and Africa regions, have decided to combine their efforts to create the Africa, Middle East, and Europe (AFMEE) IAGG e-TRIGGER programme, whose activities started in May 2023. The involvement of the Middle East, which was already involved in the presence-based courses under the auspices of the Middle East Academy of Medicine (MEAMA), chaired by Dr. Abdul Abyad, is a great opportunity for this region, which has not yet constituted an independent IAGG region.

Second, it is anticipated that a second new initiative may also come into being, as was already planned by the leaders of the Latin America Academy of Medicine of Aging (ALMA), namely the launch in 2023 of the LATAM IAGG e-TRIGGER, with teaching exclusively in Spanish. This third programme, covering yet another time zone of the world, will be based on the same training concepts and will meet the specific and culturally adapted training needs of all healthcare professionals from the 24 countries already engaged in the ALMA.

## Conclusions

With the emergence of the COVID-19 pandemic, the thirty-year history of training future leaders in the care of older adults forced us to rethink our teaching methods and create a new online model of training called the IAGG e-TRIGGER programme (TRaining In Geriatrics and GERontology). The new model is based on the flipped learning and ECHO method and enables close and interactive participation in the training moderated by experts (tutors), and animated by young academic leaders in geriatric medicine or gerontology (scholars) and trainees (promising students hoping to further their career in the care of old adults). The Asia IAGG e-TRIGGER was immediately recognized and applauded as a valuable way of facing the urgent lack of skilled healthcare professionals in geriatric medicine and the various and complementary fields of gerontology. The success of the Asia course, now named ASIO (Asia-Oceania IAGG e-TRIGGER) continues unabated. The new Africa-Middle East-Europe (AFMEE) course will cover a second world region, where there is a similar need for trained professionals in geriatrics/gerontology, and where ageing is also accelerating. The third IAGG e-TRIGGER programme plans to target Central and South America, whose training needs are already well provided for by the excellent activities of the Latin America Academy for Medicine of Aging (ALMA). This worldwide training initiative, which will soon cover three large areas of the world, represents a unique and major effort of the IAGG-World in the training of healthcare professionals in the field of ageing care, and its positive contribution will be felt by citizens all over the world.

## Data Availability

Data sharing not applicable to this article as no datasets were generated or analysed in the submitted work.
